# Non-Communicable Diseases and Rare Diseases: A Current and Future Public Health Challenge within Pediatrics

**DOI:** 10.3390/children9101491

**Published:** 2022-09-28

**Authors:** Valeria Calcaterra, Gianvincenzo Zuccotti

**Affiliations:** 1 Department of Internal Medicine, University of Pavia, 27100 Pavia, Italy; 2 Pediatric Department, “Vittore Buzzi” Children’s Hospital, 20154 Milan, Italy; 3 Department of Biomedical and Clinical Science, University of Milano, 20157 Milano, Italy

The global burden of non-communicable diseases (NCDs) and rare diseases constitutes a current and future public health challenge within pediatrics [1,2,3,4]. The period of growth and development during childhood has a crucial impact on future health and quality of life during adulthood [5]. Children are the cornerstone of life course approaches for the prevention, management, and treatment of NCDs and rare diseases, and these approaches represent a “golden window of opportunity” to improve and promote every patient’s right to health.

NCDs are a set of *chronic diseases* that are not transmitted from person to person, have a long duration, have generally slow progression, and are rarely completely curable, presenting a significant burden on individuals, communities and economic resources [1,6]. The main NCDs are cardiovascular diseases, cancers, respiratory diseases and diabetes; however, the term “NCDs” has been extended to cover a wide range of health problems, such as hepatic, renal and gastrointestinal problems; endocrine, hematological and neurological disorders; skin disorders; genetic conditions; trauma; mental disorders; and disabilities [1,2,3].

NCDs result from interactions between genetic, physiological, environmental and behavioral factors (e.g., physical inactivity, unhealthy diet, obesity, and the consumption of tobacco or alcohol) and are often characterized by interconnected cause–effect chains [7]. NCDs tend to manifest in adulthood; however, their origins lie in conditions and behaviors that were adopted during childhood and adolescence. Additionally, the risk of NCDs can be established as early as in the womb, as the health status of women before and during pregnancy influences the susceptibility of their children to NCDs in later life [8,9]. As supported by the “developmental origins of health and disease” hypothesis, environmental conditions during fetal and early postnatal development can impact a person’s lifelong health and capacity, with persistent effects on growth, structure and metabolism [10]. Thus, it is imperative that children’s issues are an integral part of the global discourse about NCDs [11]. The early prevention of NCDs and their complications, starting from infancy and childhood, should be at the heart of all health policies and management [1].

Similarly, the burden of rare diseases that develop in childhood is also significant. Rare diseases are defined as those that affect less than 200,000 individuals in the US or less than 1 in 2000 people in Europe [4,12]. There are over 6000 known rare diseases, and in 80% of cases, they are of genetic origins. Rare diseases involve the musculoskeletal, respiratory, immune, nervous, cardiovascular, hematologic, urinary, endocrine and metabolic systems. Nearly 50–75% of rare diseases begin in childhood, which deserves special attention as the needs of children who are affected by rare diseases should be considered separately from those of adults [4]. The peculiarity of anatomical, physiological, cognitive, social and emotional features is crucial to consider in order to identify the best interventions for the wellbeing of these patients. Dedicated management and tailored care for children affected by these diseases could improve their present and future quality of life. Improvements in access to newborn screening and the facilitation of a coordinated research effort could help to design more effective interventions and to reduce recurrence rates through parental counseling [4]. Research into diagnoses, the development of orphan drugs, and emerging technologies from the perspective of rare diseases are needed to implement appropriate care for these patients.

Children with NCDs and rare diseases often face lifelong challenges in managing and treating their conditions [12]. “Vulnerable” children are more likely to be marginalized from access to healthcare and are at higher risks for poor outcomes. Healthcare systems must adopt appropriate responses to the growing burden of these diseases [13]. The treatment of NCDs and rare diseases requires close interactions between experts from different medical disciplines in interdisciplinary approaches. Children can die from treatable NCDs (such as heart disease, type 1 diabetes, asthma and leukemia) or rare diseases when health promotion and specified care are not provided [13]. The rising trends in NCDs and rare diseases require child-centered and sustained efforts to prevent and screen these diseases and to improve the quality of life and chances of survival among children who are affected by these diseases. The evolution of personalized and precision medicine is crucial for understanding the pathogenic mechanisms of diseases and for ameliorating the management and monitoring of childhood diseases. New, advanced and complementary therapeutic approaches within different areas of pediatric care could solve critical issues for children with NCDs and rare diseases [14].

This Special Issue, “Non-communicable diseases and rare diseases in pediatrics: management, treatment and prevention strategies”, provides readers with an up-to-date overview of important progress in the diagnosis, management, treatment and prevention strategies for children with NCDs and rare diseases, as well as any complications that they may face, in order to identify and address the special healthcare needs of children who are affected by these diseases.

## Data Availability

Not applicable.
